# Migration and the epidemiological transition: insights from the Agincourt sub-district of northeast South Africa

**DOI:** 10.3402/gha.v7.23514

**Published:** 2014-05-15

**Authors:** Mark A. Collinson, Michael J. White, Philippe Bocquier, Stephen T. McGarvey, Sulaimon A. Afolabi, Samuel J. Clark, Kathleen Kahn, Stephen M. Tollman

**Affiliations:** 1 MRC/Wits Rural Public Health and Health Transitions Research Unit (Agincourt), School of Public Health, Faculty of Health Sciences, University of the Witwatersrand, Johannesburg, South Africa; 2 Centre for Global Health Research, Division of Epidemiology and Global Health, Department of Public Health and Clinical Medicine, Umeå University, Umeå, Sweden; 3 INDEPTH Network, Accra, Ghana; 4 Department of Sociology, Population Studies and Training Center, Brown University, Providence, RI, USA; 5 Centre de recherche en démographie et sociétés, Université Catholique de Louvain, Louvain-la-Neuve, Belgium; 6 Brown University School of Public Health, International Health Institute, Brown University, Providence, RI, USA; 7 Department of Sociology, University of Washington, Seattle, USA; 8 Institute of Behavioral Science (IBS), University of Colorado at Boulder, Boulder, CO, USA

**Keywords:** migration, temporary migration, mortality, epidemiological transition, South Africa, Agincourt, health and demographic surveillance

## Abstract

**Background:**

Migration and urbanization are central to sustainable development and health, but data on temporal trends in defined populations are scarce. Healthy men and women migrate because opportunities for employment and betterment are not equally distributed geographically. The disruption can result in unhealthy exposures and environments and income returns for the origin household.

**Objectives:**

The objectives of the paper are to describe the patterns, levels, and trends of temporary migration in rural northeast South Africa; the mortality trends by cause category over the period 2000–2011; and the associations between temporary migration and mortality by broad cause of death categories.

**Method:**

Longitudinal, Agincourt Health and Demographic Surveillance System data are used in a continuous, survival time, competing-risk model.

**Findings:**

In rural, northeast South Africa, temporary migration, which involves migrants relocating mainly for work purposes and remaining linked to the rural household, is more important than age and sex in explaining variations in mortality, whatever the cause. In this setting, the changing relationship between temporary migration and communicable disease mortality is primarily affected by reduced exposure of the migrant to unhealthy conditions. The study suggests that the changing relationship between temporary migration and non-communicable disease mortality is mainly affected by increased livelihood benefits of longer duration migration.

**Conclusion:**

Since temporary migration is not associated with communicable diseases only, public health policies should account for population mobility whatever the targeted health risk. There is a need to strengthen the rural health care system, because migrants tend to return to the rural households when they need health care.

Migration and urbanization are strongly implicated in the intertwined demographic and health transitions. These aspects of population distribution are associated with (even help drive) the demographic transition. At the same time the geographic redistribution of persons, chiefly from rural to urban areas, exposes them to new health regimes, and the stress of relocation itself may introduce its own health consequences. Furthermore, in a mobile world, the continued circulation of persons between origin and destination may serve to expedite the spread of disease. At the same time our understanding of the interconnection between population redistribution and health dynamics is poorly understood. Analysts and policy-makers often know only the broad sweep of net changes across gross geography; fine-grained, longitudinal information about movement – both with respect to time and to place – is often unavailable.

In South Africa, circular labor migration was a cornerstone of apartheid policies and accompanied national economic and industrial growth, leaving legacies that still shape contemporary opportunities for rural South Africans (1–3). Mineral discoveries of the late 19th century led to rapid development of the mining industry and parallel industrialization. For political and economic reasons, a ‘Bantustan’ system was developed which restructured the settlement patterns and livelihood strategies of the native African population to provide necessary labor, while forcing unemployed family members to remain in densely settled, rural, and peri-urban areas (2, 4). Thus, circular labor migration played a key role in the success of the national economy, while placing a heavy burden on rural households (5, 6). Nowadays, temporary labor migration remains a prime strategy used by rural households to kick back against poverty (2, 7). Gold mining has been a key employment sector, and recent investigations have revealed a significant epidemic of silicosis in former mine workers (8). Other health impacts of the migrant labor system on migrants and the households left behind have been the largest HIV/AIDS epidemic in the world (9–11) and linked to this an intractable tuberculosis (TB) epidemic (12).

Our use of the Agincourt Health and Demographic Surveillance System in the rural northeast of South Africa, located in a former ‘Bantustan’ area, help us better understand the foundations of the relationship between migration – and urbanization, since the migration is often to urban areas – and mortality. Our rural district is economically dependent on opportunities in the major metropolitan areas of Johannesburg and Pretoria, and mines close to these cities, about 500 km away. Our approach enables us to take a detailed look at a small, well-defined population, where an exhaustive population register has kept a record of residents, migratory behavior, and mortality, with the sub-district boundary acting as a migration-defining boundary (13).

In this paper, we examine several key patterns of demographic dynamics with respect to this rural sub-district population, including the patterns, levels, and trends in temporary migration linking rural and urban areas, as well as the mortality trends by sex for the rural sub-district in the 12-year period, 2000–2011. We further provide regression-based analyses of the relationship between temporary migration and mortality in this population.

## Literature review

### The epidemiological and demographic transitions

Two paradigmatic transitions have offered considerable insight into change within populations over time: the epidemiological transition and the demographic transition. Both are the subject of extensive writing, which includes critiques of these as contemporary paradigms. We here provide a brief exposition of these paradigms (given widespread familiarity) and we concentrate our discussion on the connection between the two. Note that while the ‘epidemiological transition’ is the phrase that has long held sway, contemporary writing also makes reference to a broader ‘health transition’ (14, 15). We consider the epidemiological transition (with its focus on causes of morbidity and mortality) to be embedded within a health transition (which subsumes the former and also considers risk factors, and other characteristics). Recent writing on the global burden of disease (GBD) has expanded from concerns about mortality to risk factors related to morbidity and loss of healthy years of life (16, 17). We regard the epidemiological transition in its conventional sense, generally the transition from the acute communicable diseases (CDs) of poverty to the chronic non-communicable diseases (NCDs) of high-income societies. The classic exposition of this transition is due to Omran (18), although there have been numerous repositionings, extensions, and analyses both within and across low- and middle-income countries (19–21).

The second key transition of concern here is the demographic transition. Like the epidemiological transition, the demographic transition is paradigmatic and has experienced likewise a number of repositionings, and extensions (22–24). As stated most simply the demographic transition is a movement from a regime of high mortality and high fertility to one of low fertility and low mortality. The epidemiological transition is embedded within the demographic transition through mortality reduction. Population growth rates are low or near-nil at the beginning and end of the transition, while population growth can be substantial (as, conventionally, mortality decline precedes fertility decline) in the interim phase. While the basic demographic transition is a highly structured paradigm, variations in the relationship between mortality and fertility trends across time and space have important implications for age structure and economic development, and it is linked to a number of other phenomena, including urbanization (23).

Public health discussion once presumed an inexorable parallel between economic development and health transitions, but changes in both technology and disease regimes have challenged this paradigm. As populations move from the diseases of poverty to those of wealth, this rise in NCDs will have profound impacts on health care systems of low- and middle-income countries (LMIC) (25, 26). Many African populations already manifest risk factors for NCDs, despite remaining classified well within the World Bank low-income category. NCDs have already reached to a level of about one-quarter of all mortality in the WHO Africa Region (27). Of particular note is the need to understand the heterogeneity in the dual burden of both CDs and NCDs, a public health problem of rising concern (28–32). Thus, the association between development indicators and the health transition is different at the national and local levels, and we cannot consider NCDs to be solely conditions of national affluence (21). The disjuncture between the conventional development and population health paradigm and the newly developing and heterogeneous health transition is particularly acute and consequential for Africa, where the burdens of poverty, HIV, and other diseases are sharply felt.

The recently updated GBD 2010 study (Global Burden of Disease) points quite clearly to the dual burden currently experienced in developing settings, linking these to leading risk factors. For sub-Saharan Africa, the GBD 2010 study finds high (and thus adverse impact) rankings for high blood pressure and tobacco use, while also finding high rankings for suboptimal breastfeeding, inadequate sanitation, and iron deficiency. More specifically and pertinently for our sub-region of interest, the GBD team writes ‘In 2010, alcohol use was the leading risk factor in southern sub-Saharan Africa, followed by high blood pressure and high body-mass index …’^17^.

The contemporary pace of the epidemiological (or health) transition argues further for consideration of its relationship with migration, urbanization, and the overall demographic transition. The demographic transition in Sweden occurred on a time scale of over a century. In more recently developing parts of the world the demographic transition has proceeded more swiftly. In the Southern African region as a whole, UN estimates indicate that the overall growth rate declined from 2.41% annually in the 1950s to 0.9% annually in the 2000–2010 interval. Even more pointedly, the demographic transition (as seen through the key driver of fertility) has been evident in the Agincourt study population, where the total fertility rate (TFR) averaged 6.0 in 1979 has declined to 2.3 by 2004 (33). Despite the rapid overall transition, significant differentials exist by African geographic sub-region and urbanization level (34).

Long-standing CDs still matter much. For contemporary African children, 75% of the burden of disease is accounted for by malaria, diarrheal disease, respiratory infections, and other CDs (17, 35). However, the dual burden of disease is already manifest: sub-Saharan Africa exhibits death rates from cardiovascular disease (CVD) parallel to LMIC South Asia or Central Asia (35). This phenomenon can be seen in all contemporary developing settings but is especially pertinent to sub-Saharan Africa (30, 36, 37).

### Migration, urbanization, and health

The majority of the world's population lives in urban areas now. Even in sub-Saharan Africa, some 37% of the population resides in urban areas (38). While the urbanization associated with economic growth is likely to raise average levels of well-being, there has always been some concern about the potentially deleterious effects of urbanization on health (39, 40). This concern takes several forms, but central is the shift in exposure regime. Whereas in rural areas certain endemic vector-borne diseases drive the burden of disease, in urban areas these are reduced in incidence (not eliminated!) but are supplemented with concerns that accompany issues of sanitation in dense settings, exposure to air pollution, and the like. Added to risk in urban settings are the diseases of more sedentary lifestyles and purchased foods concomitant with rising incomes and CVDs and the low health literacy among citizens and clinicians about NCDs such as hypertension and type 2 diabetes. For instance, in a broad review article on CVD (41), Yusuf and co-authors strongly implicate urbanization in developing societies. With urbanization (or migration to Western environments), there is a marked increase in consumption of energy rich foods, a decrease in energy expenditure (through less physical activity) and a loss of the traditional social support. While these behavioral and health trends hold broadly – and while they clearly implicate migration and urbanization – the timing and features of the relationship are less clearly identified. Thus, both individual level exposures and health system unpreparedness in some urban neighborhoods can serve to exacerbate the rise of NCDs. Although, overcrowded conditions that accompany urbanization (and particularly urban poverty) may increase risk of infectious diseases, other aspects of resettlement to urban settings may increase the risk of exposure to environmental hazards, in turn linked to the potential for respiratory disease and diarrhea (42).

In addition to its role in urbanization, migration itself is further linked to health. Migration can be stressful, just as is the case with many major life transitions. Migration is also implicated in the transmission of human disease, and infected individuals carry disease from one location to another. Nowhere is this more in evidence and of concern that with the spread of HIV in southern Africa, a phenomenon very relevant to our analysis here (11, 43). Even though migration may disrupt or even sever residential patterns, often the networks, and sharing of resources – most typically in the form of remittances – persist. These flows of monetary and non-monetary resources are also quite relevant to our population. Although some public health writing on migration and urbanization discusses the severing of ties and loss of social support that may accompany urbanward migration (41, 42), studies within the migration field often take note of the variety of strategies that migrants undertake to adapt in the destination and/or remain tied to the origin community through monetary remittances or other forms of communication (44, 45). At the same time, the urban setting may provide access to alternative social networks or more specific health services that could beneficial for health (46, 47). Thus, the net impact of migration could be positive or negative, and empirical analysis, such as we conduct in this paper, is necessary to sort out the balance.

The contribution of migration to urban growth is low in Africa compared to other developing regions, in part due to the highly circular nature of rural–urban migration on the continent (48). This means that there is highly prevalent back-and-forth mobility of young adults freeing themselves from constraints and poor opportunities in rural areas and joining the unpredictable urban setting in search of better livelihood opportunities (40). For many urban centers in sub-Saharan Africa, there is evidence of increasing levels of circular migration, which has reduced the contribution of in-migration to urban growth (48). As our discussion below indicates, we observe a population with a high degree of circulation.

### Conceptual framework

**Diagram 1:**
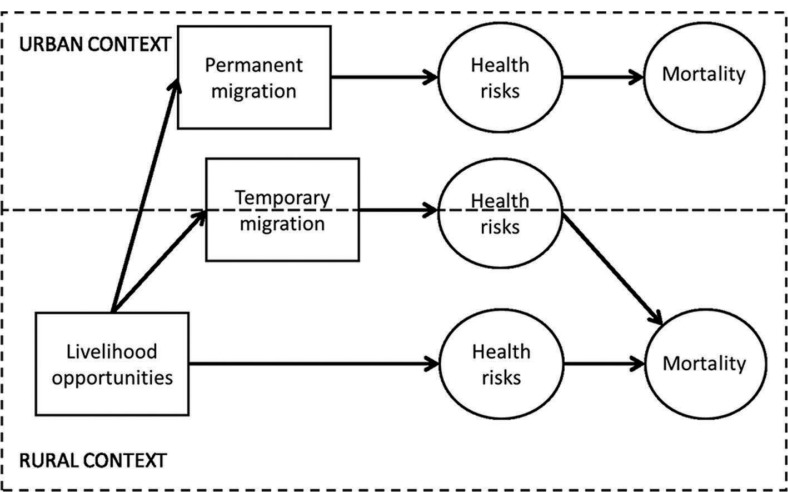
Migration–health conceptual diagram.

The preceding literature review incites us to consider circular, that is, temporary rural–urban, migration in a different way than permanent migration to urban areas. Diagram 1 shows that although the two types of migrations are motivated by the (lack of) livelihoods opportunities, the latter leads to a permanent, often definitive change of environment, while the former exposes to health risks at both origin and destination (hence boxes placed on the border of rural and urban areas). Despite their dual residence temporary migrants’ deaths usually happen in rural areas because these migrants are attached to rural households. In other words, the burden of disease does not reflect the exposure of temporary migrants, which has implications for policies and programs. Places which expose migrants to higher risks are not necessarily places that support most of the consequences on health.

The diagram is not meant to depict all possible moves (including in-migration and return migration) and corresponding health risks and the paper does not attempt to compare health outcomes of in- and out-migrants with non-migrants or temporary migrants. It will thus not address issues of selection by migration. Consequently, the diagram is meant to pinpoint the peculiar health situation of temporary migrants sitting astride urban and rural areas and depict a typical situation when migrants cross a rural–urban border. This can be extended to more complex situations encountered in South Africa where migrants go and work in (urban) mining locations or industrial farms. ‘Urban areas’ in the diagram could also be named ‘labor markets’, and ‘rural areas’ be named ‘subsistence economies’.

**Diagram 2:**
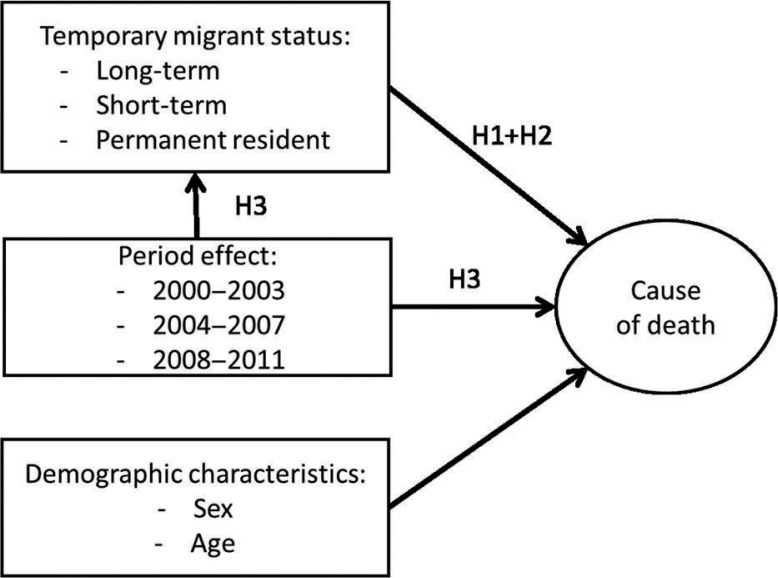
Rural migration–health empirical model.

The present empirical study focuses on the lower part of the diagram, that is, on rural areas. Diagram 2 is an empirical translation of diagram 1 and summarizes the variables available for analysis in Agincourt Health and Demographic Surveillance System (HDSS) that will serve to test hypotheses on the migration–mortality relationship. Temporary migration exposure is our main independent variable. The evolution of livelihood opportunities (including changing health system context) is captured through period effect and its interaction with migration exposure. The control variables are the demographic characteristics (age and sex). While CDs are usually associated with younger adults and NCDs with older adults, the disease prevalence by age is by no means exclusively dichotomous and mortality from NCDs occurs at increasingly younger ages. To accommodate this we used age as the temporal dimension in the regressions, which creates an age standardization throughout the analysis.

Diagram 2 also indicates which causal relationships the following hypotheses are testing:

H1 Temporary migration, net of demographic characteristics, predicts higher rates of overall mortality as compared to permanent residents in rural areas. Longer exposure to health risks in destination areas should lead to higher mortality rates.

H2 The second hypothesis is embedded in the first: Temporary migration is less strongly associated with the relative risk of death from NCD. CDs should be the highest health risks that circular migrants are subjected to.

H3 Both overall mortality and the impact of temporary migration on mortality have declined over time, as temporary migrants and permanent residents have experienced better livelihood opportunities, including an increase in health outreach, especially antiretroviral treatment (ART) roll-out.

## Methods

### Health and demographic surveillance system

The Agincourt HDSS was established in 1992, and it was designed to capture the health, sociodemographic, and other information of all the residents of Agincourt, a sub-district of the Bushbuckridge municipality situated in rural north-eastern part of South Africa. Agincourt encompasses a geographical area of about 420 km^2^ where a pre-dominantly Shangaan-speaking black South African population reside. The surveillance started with a baseline census in 1992 and was followed by annual updates of the initial records in the subsequent years. The initial population was about 70,000 individuals residing in 21 villages. In 2007, the study population was expanded by increasing the number of villages under surveillance to 27, with an expanded population of about 90,000 (49). This paper uses data collected between 2000 and 2011. In addition to collecting information about births, death, migrations, unions, and household membership, specialized census modules are employed to capture different socioeconomic data in each annual census round. Modules are repeated with a certain period to provide detail about the dynamics of key phenomena that influence health and demographic patterns (49, 50).

### Migration methods

Migration is recorded in one of two ways in the HDSS depending on the report given by the household respondent. If a person enters or leaves a household with a permanent intention, it is recorded as a permanent in- or out-migration event with details captured, such as date of move, origin or destination place, and reason for the move. Permanent residents include all non-migrant residents who did not move over the 2000–2011 period as well as in-migrants from the time they start residing in the site (being left-censored) and out-migrants until they move out of the site (being right-censored). Individuals may be both in-migrating and out-migrating over the period. It is important to note that in this study, we do not attempt to analyze the differential mortality outcomes of the sub-categories of permanent residents. Neither do we try to analyze the effect of in- or out-migration as an event since that would require the population at risk at both ends of the migration streams. Rather, we analyze outcomes of permanent residents against those of temporary migrants exposed to both origin and destination environments.

**Fig. 1:**
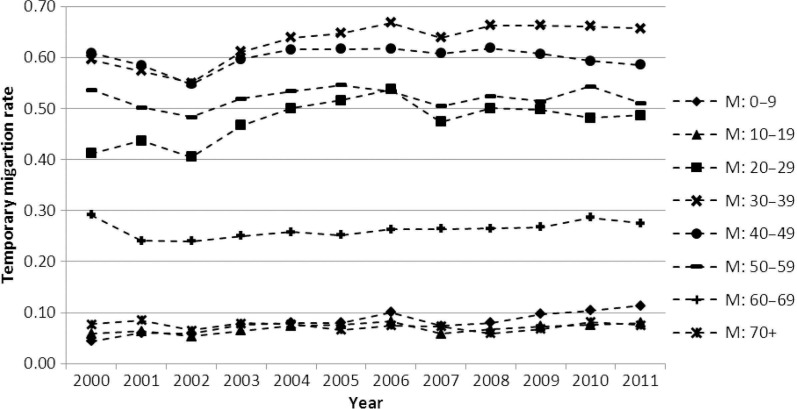
Male temporary migration rate trend by calendar year and 10-year age groups, Agincourt, 2000–2011.

**Fig. 2:**
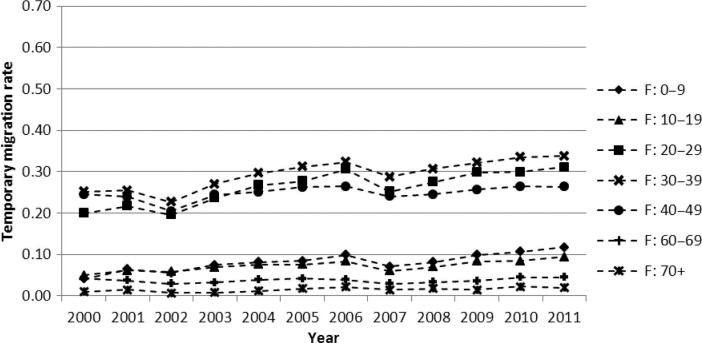
Female temporary migration rate trend by calendar year and 10-year age groups, Agincourt, 2000–2011.

A person is recorded as a temporary migrant if the respondent says that they exited the household with a temporary intention and now resides elsewhere in a second household. A temporary migrant continues to be a household member and is not removed from the household roster. Temporary migration status is updated annually with reference to the year preceding the household interview by asking on how many months a person was physically present (on aggregate) out of the previous 12, with 6 months or less classified as temporary migrant. Figures 1 and 2 show data obtained from this temporary migration status question. In the migration and mortality analyses, two categories of temporary migration duration are used, viz. short duration, when the completion of a temporary migrant cycle is within 3 years, and long duration, with completion of a cycle in longer than 3 years. Three years duration is an arbitrary cut-off used to discriminate between circumstantial or exploratory temporary migration (short duration) and an entrenched pattern (long duration), with the assumption that a 3 years or longer duration requires a relatively stable incentive such as a permanent employment contract, which can have different mortality consequences.

In addition to the annual temporary migration status update, there is a more comprehensive temporary migration module run every 5 years that was implemented in 2002, 2007, and 2012. The aim is to acquire more detail on the temporary migration, such as destination, reason for move, and the nature of links between the origin and destination households, including remittance transfers (49). Entry into this module requires that the definition of temporary migration status be met. This implies that from the perspective of the respondent, the intention of the migrant is to remain a member of the rural household while away and that the person was absent for at least 6 months out of the 12 months preceding the interview. The inferred intention of the migrant in the definition may seem unstable from a scientific perspective, because intentions and behaviors can change or be misreported, but in this context there has been much experience of labor migration and the notion of a non-resident household member is well understood. If a temporary migrant is deemed by a respondent to no longer have the intention of remaining a household member, then the fieldworker can permanently out-migrate the person and they are removed from the household roster. Data from the periodic temporary migration modules are used in Table 1 to highlight the link between the temporary migrant and rural household and also to validate the definition.

**Table 1:** By sex and observation year: percent of temporary migrants employed and percent of employed temporary migrants that remitted cash or another item back to the origin household

	2002		2007		2012		Total	
				
% of temporary migrants employed	*n*	%	*n*	%	*n*	%	*n*	%

Male	5,529	75	7,042	78	7,106	73	19,677	75
Female	2,021	53	2,532	52	2,745	49	7,298	51
Test sex difference		M>F		M>F		M>F		M>F
*p*		*p*=0.000		*p*=0.000		*p*=0.000		*p*=0.000

	2002		2007		2012		Total	
				
% of employed migrants that remitted	*n*	%	*n*	%	*n*	%	*n*	%

Male	3,820	69	4,437	64	4,815	68	13,072	67
Female	1,433	71	1,653	66	1,994	73	5,080	70
Test sex difference		M<F		M<F		M<F		M<F
*p*		*p*=0.065		*p*=0.01		*p*=0.000		*p*=0.000

Temporary migration as a repeatable exposure is the key independent variable used in the analysis. To understand the relative prevalence of temporary migration compared to permanent migration, the Crude Migration Rates were computed in 2006, a year in the middle of the observation period. For females, the permanent in-migration rate was 26/1,000 and the permanent out-migration rate was 30/1,000, while the temporary migration rate was 177/1,000. For males, in 2006, the permanent in-migration rate was 17/1,000, the permanent out-migration rate 22/1,000 and the temporary migration rate was 322/1,000.

### Mortality methods

Each death that occurs in the intercensal period is recorded during the annual vital events update. A second interview for a ‘verbal autopsy’ is conducted on each death by a trained lay fieldworker to establish the most probable cause of death. The interview is conducted with the closest caregiver to the deceased to establish the most prominent signs and symptoms occurring prior to death. These data are captured onto a database system and transformed into the input format for the InterVA4 analysis software that uses an algorithmic approach, calibrated by knowledge of local disease patterns, to establish the main cause of death, as well as immediate and contributing causes (51–54).

**Fig. 3:**
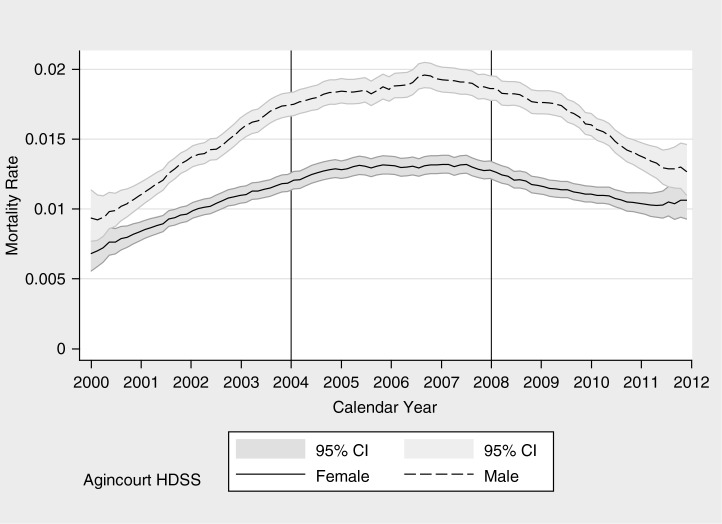
All-cause mortality rate by calendar year for males and females, Agincourt HDSS.

**Fig. 4:**
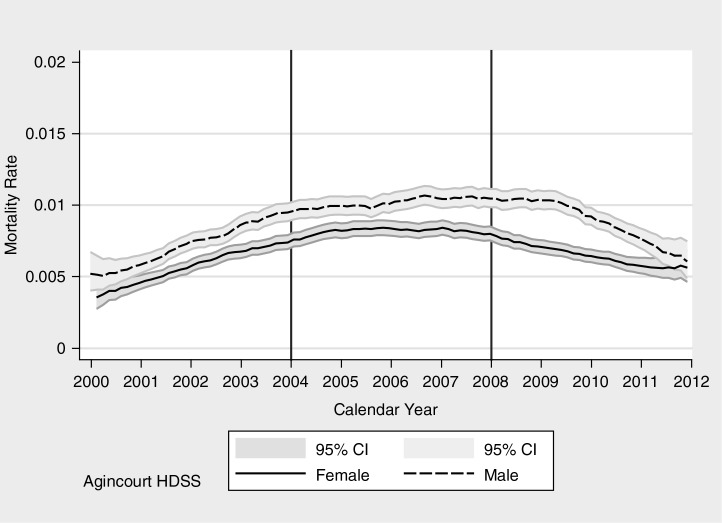
Communicable disease mortality rate by calendar year for males and females, Agincourt HDSS.

**Fig. 5:**
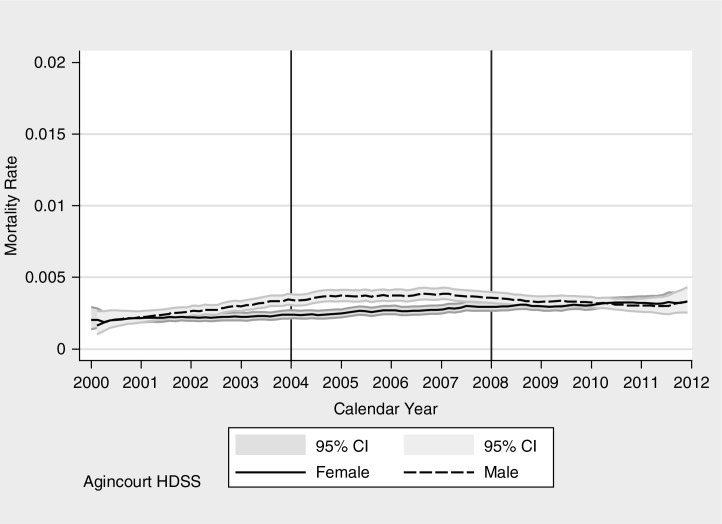
Non-communicable disease mortality rate by calendar year for males and females, Agincourt HDSS.

The broad cause of death categories used are CD, NCD, external causes, and undetermined causes. These categories are made up of constituent causes of death and the categorization is shown in a Supplementary file, where constituent causes of death are ordered by rank of frequency. For CDs, the most important three constituent causes were HIV/AIDS, pulmonary TB, and acute respiratory infections; for NCDs: chronic obstructive pulmonary disease, cardiac disease, and stroke; and for external causes of death: assault, motor vehicle accident, and intentional self-harm. The mortality trends in Figures 3 to 5 and the regression results in Tables (2–5) are from the core HDSS residence files, with residence status data merged in and residence criteria of 6 months in the origin household required to be considered part of the household. Mortality rates by cause of death are provided in a Supplementary file, giving age and sex profiles in the three analytic periods described later.

**Table 2:** Migration factors associated with death from communicable disease

	Hazard ratio (95% Confidence intervals); death by communicable disease		
	
Variables	2000–2003	2004–2007	2008–2011
Sex: male vs. female	1.65*** (1.46–1.87)	1.74*** (1.58–1.93)	2.27*** (2.03–2.54)
Short duration temporary migrant vs. not	2.53*** (1.87–3.41)	1.15 (0.71–1.87)	1.30 (0.73–2.31)
Long duration temporary migrant vs. not	2.70*** (2.33–3.14)	1.65*** (1.48–1.85)	0.78*** (0.68–0.88)
Observations	52,680	52,543	55,847
Wald Chi-square	330.7	245.3	214.7
Log likelihood	−7,719	−11,836	−10,273
Subjects	48,661	49,573	53,271
Failures	1,101	1,660	1,436

*** *p*<0.01. Comparing temporary migrants with permanent residents, controlling for age and sex.

**Table 3:** Factors associated with death from non-communicable disease, by migration category and period, controlling for age and sex

	Hazard ratio (95% Confidence intervals); death by non-communicable disease		
	
Variables	2000–2003	2004–2007	2008–2011
Sex: male vs. female	1.50*** (1.25–1.81)	2.06*** (1.76–2.42)	1.81*** (1.54–2.12)
Short duration temporary migrant vs. not	1.88** (1.05–3.35)	1.70 (0.81–3.59)	1.18 (0.37–3.76)
Long duration temporary migrant vs. not	1.55*** (1.17–2.04)	1.08 (0.87–1.33)	0.60*** (0.48–0.74)
Observations	52,680	52,543	55,847
Wald Chi-square	41.23	90.19	60.68
Log likelihood	−3,195	−4,283	−4,600
Subjects	48,661	49,573	53,271
Failures	493	645	698

*** *p*<0.01

** *p*<0.05.

**Table 4:** Hazard ratios showing the association of factors with death from an external cause by period and migration category, controlling for age and sex

	Hazard ratio (95% Confidence intervals); death by external causes		
	
Variables	2000–2003	2004–2007	2008–2011
Sex: male vs. female	4.53*** (3.04–6.75)	5.46*** (3.78–7.89)	5.18*** (3.56–7.55)
Short duration temporary migrant vs. not	9.50*** (5.47–16.49)	3.30** (1.32–8.28)	2.24 (0.69–7.24)
Long duration temporary migrant vs. not	5.78*** (3.82–8.74)	3.04*** (2.16–4.29)	1.45** (1.00–2.10)
Observations	52,680	52,543	55,847
Wald Chi-square	205.6	135.5	85.82
Log likelihood	−993.0	−1,165	−1,124
Subjects	48,661	49,573	53,271
Failures	147	166	153

***p<0.01

**p<0.05.

**Table 5:** Factors associated with death from unspecified cause of death, by period and migration status, controlling for age and sex

	Hazard ratio (95% Confidence intervals); death by unspecified causes		
	
Variables	2000–2003	2004–2007	2008–2011
Sex: male vs. female	1.40* (0.94–2.07)	1.63*** (1.15–2.32)	2.40*** (1.59–3.63)
Short duration temporary migrant vs. not	4.00*** (1.73–9.24)	3.93** (1.23–12.53)	1.98 (0.29–13.78)
Long duration temporary migrant vs. not	3.01*** (1.86–4.88)	2.75*** (1.85–4.07)	1.33 (0.85–2.08)
Observations	52,680	52,543	55,847
Wald Chi-square	38.28	40.22	21.05
Log likelihood	−751.2	−892.8	−649.0
Subjects	48,661	49,573	53,271
Failures	107	126	92

*** *p*<0.01

** *p*<0.05

* *p*<0.1.

### Migration and mortality analysis

We examine migration and mortality using a continuous, survival time, competing-risk model which estimates the sub-hazard ratios of migration exposures as related to mortality outcomes, comparing migrant with non-migrant categories, after controlling for the age and sex structure of the population. Given that dates are recorded to the day, a continuous-time model is preferred to discrete-time model, which is less precise because of ties, that is, when the order of censoring and event is not known within a time interval. Also, a continuous-time model makes it easier to handle large datasets since dates are recorded only when there is a change in value. To prepare the data, we constructed a residency file, whereby each individual has a sequential record kept of each demographic event that occurred to them. Events include in- or out-migrations, the date of becoming a temporary migrant, and the date of ending a period as a temporary migrant, as well as births or deaths, with deaths classified by probable main cause. Individuals were excluded from the regressions if they spent less than 6 months per year in the study population. The biographical files were censored at the end of the observation period on January 1, 2012. In the regression models, age is the temporal dimension and sex is included as a co-variate, so all the models control for age and sex. Migration is differentiated by short- and long-term duration. We did not include other controls because the analyses are already complex.

Competing risk models are used to predict mortality by cause of death categories and explore the extent to which short and long duration temporary migrations are linked to mortality by cause category. The Fine and Gray competing risk model is preferred to the usual Cox model, because it does not make the assumption of independent causes of death (55). Models are run for different cause of death categories in a competing risk model so that each cause of death is treated as sub-category of overall mortality and not as ordinary right-censoring for other causes. Models are also run for each of the three periods used in the mortality models: 2000–2003, 2004–2007, and 2008–2011. The choice of these periods reflects the overall non-linear mortality trends, while preserving enough statistical power for low mortality risk when conducting separate regression analyses by period. In the first period, the mortality trend is increasing; in the middle period the trend flattens; and in the third period, the mortality trend is declining. To account for possibly non-constant trends in the relation between migration status and mortality, we preferred to run separate models for each period rather than to introduce interaction terms between migration status and period. This is because period may have non-constant effect on mortality as well as on the relationship between migration status and mortality.

## Findings

### Temporary migration

Figures 1 and 2 show temporary migration levels and trends by sex and age group. The migration rates are quite stable over time, considering that they are independently measured each year, but there is a gradually increasing trend for some age groups. A striking feature is the high migration rate, especially for males aged 20–59. In the first half of the observation window, men of this age group were 40–60% likely to be a temporary migrant, while in the second half of the observation period they were 50–70% likely to meet the criteria for being a temporary migrant. For women, the ages 20–49 years were the most mobile, with migration levels tending to increase over the observation period, from 20 to 25% being a temporary migrant at the start of the period, to 25–35% of women being a temporary migrant at the end. Thus, for working age adults, both males and females showed an increasing likelihood of being a temporary migrant between 2000 and 2011.

Temporary migration is linked to a change in socioeconomic status in rural households in northeast South Africa (7). Table 1 shows two patterns linking migration and economic status for men and women. The upper aspect of the table shows the percent of temporary migrants that were employed. Across the observation period, approximately 75% of male temporary migrants and 50% of female temporary migrants were employed. The gender difference is highly significant with male migrants more likely to be in employment (*p*<0.000). The lower aspect of the table shows the proportion of employed temporary migrants that remitted cash or another item back to the origin household. In 2002, female employed migrants were slightly more likely than male employed migrants to remit (*p*<0.065). This difference became clearer over the decade and by 2012 it was significant (*p*<0.000) that employed female migrants were more likely to remit than employed male migrants.

### All-cause mortality

Figures 3 on all-cause mortality shows a clear pattern. The 2000–2003 period displays a constant and high increase in overall mortality; mortality is stable at high level during the 2004–2007 period; and mortality declined in the 2008–2011 period. To note, ART roll-out started systematically in 2008, with almost immediate effect on the decline of CD cause of death (11). Non-linear trends over the 2000–2011 period are essentially attributable to CDs mortality for both sexes (Fig. 4) and to NCD for males (Fig. 5), with a gradual increase in NCD mortality for females (53).

### Mortality from a CD

In Fig. 4, CD mortality trends show a rise and then a fall over the course of the study period. The scourge of AIDS and TB impacted these mortality profiles between 2000 and 2007 (11, 53) and health system made anti-retroviral treatment available in the period 2008–2011 (11).

Table 2 shows three comparable multivariate competing risk regression models, one for each period, enabling us to examine the relationship between temporary migration and CD mortality over time, with temporary migration discriminated by short duration (≤3 years) and longer duration (>3 years). There are important associations between migration and mortality from CDs. At the start of the period, temporary migration shows a particularly high risk of mortality with long duration temporary migration having 2.7 times higher risk of death than non-migrants and short duration temporary migration 2.5 times higher risk. Over time the effect lessens although it remains significantly negative for long duration temporary migrants in the middle period. In the last period, shown in the right-hand column, longer duration temporary migration has become a significantly protective factor from CD mortality. The environment for temporary migration has shown an important shift from high risk of exposure to HIV, TB, and acute respiratory infections, possibly due to the associated disruption from normal family supports and the un-healthy environments at destination, to an environment where the benefits of migration have improved, including better access to health care and relative socioeconomic well-being for migrants. By the end of the period, longer duration temporary migration has become positively associated with survival and the picture is mixed for shorter duration temporary migrants.

### Mortality from NCD

The age-adjusted level in NCD mortality increased in both sexes over the decade of observation, with male rates increasing in the earlier half of the observation period and then leveling off, and female mortality rates from NCD increasing steadily over the period (53). The Agincourt mortality profiles shown in a Supplementary file indicate that over the 8 years from 2004 to 2011, the percentage of mortality attributable to an NCD increased in all age and sex groups.

Table 3 shows three competing risk regression models, examining the relationship between temporary migration and NCD mortality over time. At the start, temporary migration shows a strong association with NCD mortality with long duration temporary migration having 1.9 times higher risk of death than non-migrants and short-duration temporary migration 1.5 times higher risk. As with CD mortality, the effect lessens over time and becomes non-significant in the middle period, and protective in the last period for long duration temporary migration. This may reflect an overall shift in the dangers associated with long-term temporary migration, from one of negative lifestyle changes, disruption, and stress at the start of the period, associated with high mortality rates from NCD for temporary migrants compared to non-migrants, to a lessening of this relationship over time, associated with an increasingly protective effect of long-term temporary migration at the end of the period.

### Migration and mortality from external causes

Temporary migration is significantly associated with mortality from external causes, including assault, motor vehicle accidents, self-harm, and other causes. The gender dimension is striking with men four to five times more likely to die from an external cause compared to women. Both short and long duration temporary migrations stand out as exposures significantly associated with increased risk of mortality from an external cause of death at the start of the decade, but the negative relationships lessen over time.

### Migration and mortality from undetermined causes

For undetermined causes of death, the numbers of cases are much fewer than the main causes of death and the general impact of migration follows a similar pattern as seen above with long duration temporary migration showing a significant mortality risk in the first two thirds of the period, which has become insignificant by the end of the period.

## Discussion

The availability of long-term demographic and health surveillance data allows us to gain insights into the trends in temporary migration and mortality and their relationship over the period 2000–2011. The results relate to patterns of urban transition in settings with high levels of temporary circular migration. Analytically, temporary migration identifies the individuals that are away for most of the time, but remain linked to the rural households of origin. The data presented show the stability and even growth of temporary migration patterns in rural, northeast South Africa. Remittance patterns are also stable and although men are more likely to migrate for employment reasons, women are increasingly likely to migrate and a half of these female migrants are also employed. In this paper, we do not explore the frequency, quantity, or size of the remittances, but the two variables, ‘percent of migrants employed’ and ‘percent of employed migrants that remit’, indicate the intrinsic economic importance of temporary migration. Furthermore, employed female temporary migrants are more likely than males to remit something back to the original household. Under apartheid, rural South African households were forced to participate in an imposed migrant labor system, but current data show the ongoing relevance of labor migration as a rural livelihood strategy for men and women.

There are features in the mortality trends presented here that are consistent with the conventional epidemiological transition in this rural sub-district. Coupled with severe mortality increases from CDs in the first half of the period, due primarily to AIDS and TB (53), mortality from NCDs has been increasing, albeit more gradually, which has led to the pattern of a dual burden of illness (17). The mortality rates from AIDS/TB started coming down from 2008/9. Although the reasons for this are not directly examined in the paper, there has been a widespread recognition that making anti-retroviral therapy available at public hospitals and health centers has played a major role (11). Thus, we see in this one setting, aspects of the conventional epidemiological transition, but equally important key aspects of the revision to that transition more in step with current thinking regarding the GBD.

Most notably our empirical work suggests that caution should be taken in not making too simplistic an association of age and geography with health outcomes. In many instances, temporary migration is more important than age and sex in explaining variations in mortality. Temporary migration is shown to have a relationship with mortality from CDs, but the relationship is not consistent over time. Notable here is that the Agincourt HDSS data are distinctly privileged in identifying and following temporary migrants; other data sources (often on which discussions of health transition are based) do not have such detail. A single interval of about a decade in length is arguably insufficient to either confirm a new pattern for an epidemiological transition or refute it. Still, the range of changes we see, included dramatic prospective shifts in health prospects due to HIV/AIDS treatment, along with the ongoing dramatic changes in population mobility and the technology of transportation and communication, argue for continued attention to the dynamics of migration and health.

We find a strong association of migration and CD mortality, especially for male temporary migrants, in the early part of the observation period. The relationship is most likely driven by a process whereby temporary labor migrants that become ill as a result of exposure outside home, may eventually die in their rural households (56). In the latter part of the observation period conditions for temporary migrants seem to have improved such that the association of temporary migration and survival becomes a positive relationship. Causes are not directly examined in the study, but three factors can play a role. First, the risk of transmission of infectious disease has possibly declined through temporary migration becoming more selective in nature and the process less stressful for the migrant. Second, temporary migrants and their origin households are more connected through mobile phone technology, which can lessen the impetus for extra-marital sexual partnerships. Third, the improved access to health care may have benefited migrants, especially by ART being made available through government and non-government programs targeting migrant workers.

The association of migration and NCD mortality shows a relationship that transitioned toward a healthier association, with temporary migration starting as a possible risk factor at the beginning of the period, but becoming more positive over time. Although we do not have causal models to directly examine risk factors in the temporary migration and mortality relationships, we have included a small interpretive device by discriminating between shorter and longer duration temporary migrants in the regressions. As explained in the literature review, there is evidence of both positive and negative health implications of migration in different settings, and similarly positive and negative implications for household socioeconomic status reported in different studies. To explore which direction our data point we can assume that longer duration temporary migrants of both sexes are more likely to have succeeded in gaining employment and thus are more likely to be a consistent wage earner. Shorter duration migrants are less likely to be consistently earning a wage. If temporary migrants’ relationship to mortality is influenced by exposure to unhealthy environments, diet and or other lifestyle factors, like alcohol and smoking, then shorter duration migrants would be exposed for shorter times and thus show less association with mortality, and, conversely, longer term migrants show more association with mortality. Although far from conclusive, our findings indicate that CD mortality has a stronger association with longer duration migration in the earlier part of the period, consistent with unhealthy exposure, which changes over the observation period into an association with better survival chances, consistent with these migration-related risks lessening over time. NCD mortality shows a somewhat different relationship with migration. A longer duration of migration is consistently less associated with mortality than shorter duration migration throughout the observation period. This may indicate that the economic benefit of longer duration temporary migration is playing a stronger positive role than unhealthy exposures playing a negative role in the relationship between migration and mortality. A similar picture is shown for external causes of death, which is even more sensitive to migration than CD or NCD mortality. In summary, risks of CD mortality may be higher for short-term migrants due to disruption and exposure, whereas for NCD mortality, the relationship to migration is driven more by the positive relation of the earnings of longer duration migrants making them less likely to succumb to a fatal illness.

While no observational study such as ours can reach the inferential conditions of a true randomized experiment (necessarily so), our design and analysis improve significantly on comparative cross-sectional analysis while also pointing the way to more sophisticated, promising data collection, and analysis for the future. It is probably true that we will not have randomized experiments regarding migrants’ status (57). Therefore, carefully crafted longitudinal studies of the redistribution of persons across geography (and associated changes in risk regime and livelihood) will be crucial for understanding the 21st century version of the epidemiological transition in sub-Saharan Africa.

Health and social policy implications arise from the importance and vitality of the rural–urban links shown in the study. When the circular nature of migration from poorer communities is not well understood, the balance between rural and urban development is potentially misconstrued. Migration dynamics that keep households economically vital are conceptually flattened through the temporary migrants being grouped into the urban category while their households of origin, to which they remain economically linked, are classified as rural.

A study limitation arises from the fact that the data are from a small sub-district in remote rural South Africa and we need to consider how representative the data are. We know the levels of circular labor migration are high, and in other areas may not be as high, but it is still valuable to examine the case study to get an empirical understanding of the trends and associations between migration and mortality. Many HDSS data systems reveal active migration patterns and comparative studies are starting to emerge (13).

Another limitation of our study is that the covariates were restricted to age, sex, period, and temporary migration status, while the outcome was restricted to large causes of death. Whereas this was useful to keep enough statistical power for our estimates, further research should aim at examining the migration–health relationship for different socioeconomic categories and also for specific diseases. This requires reconstructing individual, household and community histories consistently over the observation period in relation to migration histories. This is currently being tackled. Barring data availability, this will help target sub-populations with specific health needs.

In this study, we examined the evolution of the relationship between temporary migration status and causes of death. However, the permanent residents who form the baseline category in our study are not necessarily homogenous. A number of selective processes – some of them related to health – may change the composition of the permanent population. Previous research (56, 58) based on data from the 1990s to the mid-2000s has shown how the migrants returning sick after a long period of residence out of the site have highly contributed to reduction in life expectancy. While these studies confirm the ‘unhealthy return migrant’ hypothesis, studies are lacking to confirm in Agincourt HDSS the more famous ‘healthy migrant’ hypothesis. Future research will benefit considerably from giving more attention to the influence of individual and household migration histories on the prevalence and incidence of both communicable and NCDs, and longitudinal data-collection platforms such as the one we employ are well-suited to provide such information. Moreover, such extensive longitudinal approaches can be readily supplemented with data collection on the migrants at destination and information about social and monetary connections between origin and destination. As epidemiologists and health policy-makers demand more definitive information about the determinants of the contemporary health transition, such expanded inquiry offers considerable promise for understanding health implications of migration and urbanization.

**Main findings**

- In rural, northeast South Africa, temporary migration involving migrants that relocate mainly for work purposes and remain linked to the rural household is more important than age and sex in explaining variations in mortality, whatever the cause.
- In this setting, the changing relationship between temporary migration and communicable disease mortality is primarily affected by reduced exposure of the migrant to unhealthy conditions.
- The study suggests that the changing relationship between temporary migration and non-communicable disease mortality is mainly affected by increased livelihood benefits of longer duration migration.

**Key messages for action**

- Health care access should be expanded for migrants, especially by making patient information available to health practitioners in rural healthcare system to enhance continuity of care when a migrant returns home.
- The rural healthcare system should be improved, as migrants tend to return to the rural households when in need of health care.
- Temporary migration is associated with both non-communicable and communicable diseases; therefore, public health policies should account for population mobility whatever the targeted health risk.
